# Assessment of Fibrinogen Macromolecules Interaction with Red Blood Cells Membrane by Means of Laser Aggregometry, Flow Cytometry, and Optical Tweezers Combined with Microfluidics

**DOI:** 10.3390/biom10101448

**Published:** 2020-10-15

**Authors:** Alexey N. Semenov, Andrei E. Lugovtsov, Evgeny A. Shirshin, Boris P. Yakimov, Petr B. Ermolinskiy, Polina Y. Bikmulina, Denis S. Kudryavtsev, Peter S. Timashev, Alexei V. Muravyov, Christian Wagner, Sehyun Shin, Alexander V. Priezzhev

**Affiliations:** 1 Department of Physics, M.V. Lomonosov Moscow State University, Leninskie Gory 1/2, 119991 Moscow, Russia; anlug1@gmail.com (A.E.L.); eshirshin@gmail.com (E.A.S.); bp.jakimov@physics.msu.ru (B.P.Y.); ermolinskiy.pb15@physics.msu.ru (P.B.E.); avp2@mail.ru (A.V.P.); 2 International Laser Center, M.V. Lomonosov Moscow State University, Leninskie Gory 1/62, 119991 Moscow, Russia; 3 Institute of Spectroscopy of the Russian Academy of Sciences, Troitsk, Fizicheskaya st., 5, 108840 Moscow, Russia; 4 Department of Advanced Biomaterials, Institute for Regenerative Medicine, Sechenov University, 2-8 Trubetskaya St., 119991 Moscow, Russia; polina_bikmulina@mail.ru (P.Y.B.); timashev.peter@gmail.com (P.S.T.); 5 Shemyakin-Ovchinnikov Institute of Bioorganic Chemistry, Russian Academy of Sciences, 117997 Moscow, Russia; kudryavtsev@ibch.ru; 6 Faculty of Fundamental Medicine, K.D. Ushinskiy Yaroslavl State Pedagogical University, Respublikanskaya st. 108, 150000 Yaroslavl, Russia; alexei.47@mail.ru; 7 Campus Building E2 6, Saarland University, Experimental Physics, D-66041 Saarbrücken, Germany; c.wagner@mx.uni-saarland.de; 8 University of Luxembourg, Physics and Materials Science Research Unit, L-1511 Luxembourg, Luxembourg; 9 School of Mechanical Engineering, Korea University, Seoul, Anam-dong, Seongbuk-gu 02841, Korea; lexerdshin@korea.ac.kr

**Keywords:** fibrinogen macromolecules, RBC membrane, optical (laser) tweezers, flow cytometry, glycoproteins IIbIIIa inhibition, microfluidics

## Abstract

An elevated concentration of fibrinogen in blood is a significant risk factor during many pathological diseases, as it leads to an increase in red blood cells (RBC) aggregation, resulting in hemorheological disorders. Despite the biomedical importance, the mechanisms of fibrinogen-induced RBC aggregation are still debatable. One of the discussed models is the non-specific adsorption of fibrinogen macromolecules onto the RBC membrane, leading to the cells bridging in aggregates. However, recent works point to the specific character of the interaction between fibrinogen and the RBC membrane. Fibrinogen is the major physiological ligand of glycoproteins receptors IIbIIIa (GPIIbIIIa or αIIββ3 or CD41/CD61). Inhibitors of GPIIbIIIa are widely used in clinics for the treatment of various cardiovascular diseases as antiplatelets agents preventing the platelets’ aggregation. However, the effects of GPIIbIIIa inhibition on RBC aggregation are not sufficiently well studied. The objective of the present work was the complex multimodal in vitro study of the interaction between fibrinogen and the RBC membrane, revealing the role of GPIIbIIIa in the specificity of binding of fibrinogen by the RBC membrane and its involvement in the cells’ aggregation process. We demonstrate that GPIIbIIIa inhibition leads to a significant decrease in the adsorption of fibrinogen macromolecules onto the membrane, resulting in the reduction of RBC aggregation. We show that the mechanisms underlying these effects are governed by a decrease in the bridging components of RBC aggregation forces.

## 1. Introduction

The multicomponent blood system is responsible for the stability of hemodynamic processes and high effectiveness of microcirculation [1,2,3]. The reversible aggregation of red blood cells (RBC) is crucial for regulating the blood viscosity changes at low shear stress and hydrodynamic resistance in blood circulation [4,5,6], while coagulation (clotting) serves as a governing mechanism of bleeding prevention [7]. Both of these processes involve fibrinogen, a blood plasma protein, which is a dimeric molecule composed of pairs of α, β, and γ chains that are folded into a three-domain nodular structure [8,9]. Many pathophysiological processes, which are characterized by an elevated concentration of fibrinogen in blood plasma, are accompanied with hemorheological disorders caused by alterations of RBC aggregation [10,11]. In particular, increased fibrinogen concentration at the acute phase reaction during inflammation is directly related to the observed increases in RBC aggregation [12,13]. Enhanced fibrinogen-induced RBC aggregation occurs in a variety of infectious diseases [14,15] and genetic disorders [16]. Abnormalities in RBC aggregation also accompany cardiovascular diseases such as arterial hypertension [17,18], atherosclerosis [19], cardiac infarction, and ischemia [20], as well as during metabolic disorders such as diabetes mellitus [21,22]. During COVID-19 infection, pathological levels of fibrin and fibrinogen result in elevated membrane fragility and the pathological elasticity of erythrocytes [23]. Therefore, a complex study of the interaction between fibrinogen molecules and RBC will allow developing new approaches of the targeted treatment of hemorheological disfunctions caused by an evaluated fibrinogen-induced aggregation of RBC.

Despite their rheological significance, the mechanisms of fibrinogen-induced RBC aggregation are still not clearly investigated [24,25]. One of the discussed models is the adsorption of fibrinogen macromolecules onto RBC membrane, leading to the cells’ bridging during interaction and resulting in the formation of RBC aggregates called “rouleaux”. Macromolecules adsorption can be determined by factors of specific and non-specific interaction. The non-specific binding of blood plasma proteins to the surface of the RBC membrane is a general phenomenon that is governed by ionic, van der Waals, hydrogen, and hydrophobic interactions [26]. However, in the last few decades, a number of works reported on the existence of the fibrinogen receptors on the RBC membrane, pointing to the specific interactions between RBC and fibrinogen [27]. In the early work of Maeda et al. [28], the authors reported that the possible fragment of fibrinogen responsible for the interaction with RBC is located in its α-chain, potentially pointing to glycoproteins as possible specific receptors. The authors of the paper [29] reported on the existence of glycoproteins-like receptors on RBC membranes and connected it with the necessity of RBC adhesion on the endothelium. In addition, it was demonstrated in vitro [30] that the presence of the Arg-Gly-Asp-Ser (RGDS) peptide reduced the fibrinogen-induced aggregation of intact erythrocytes of rats. In [31], force spectroscopy measurements using an atomic force microscope demonstrated an interaction between fibrinogen and integrin-related receptor. In [32], the authors provided results in support of a specific and age-dependent interaction of soluble fibrinogen with human RBC membrane, suggesting that the CD47 complex can be the receptor. The suppressing effect of common GPIIbIIIa inhibitors (including antibodies and low molecular weight derivatives) on RBC aggregation in static conditions was investigated [33]. Glycoproteins inhibition performed by the authors led to the decrease in the parameters of RBC aggregation, although not completely abolishing it. In the work of the same authors [34], the implementation of optical techniques revealed that the GPIIbIIIa agonist monafram (F(ab’)2 fragment of humanized form of the murine monoclonal antibody) decelerated the early phase of RBC aggregation.

GPIIbIIIa is a member of the membrane integrins family of adhesion receptors, representing itself as a calcium-dependent heterodimer, and it is a receptor of soluble form of fibrinogen on the platelets membrane that is responsible for their aggregation during clotting [35,36]. GPIIbIIIa are extensively studied as the main target for antithrombotic therapy [37,38,39]. GPIIbIIIa inhibitors have been used as adjuncts for primary percutaneous coronary intervention for many years [40]. The effectiveness of GPIIbIIIa inhibitors was reported in reducing mortality in diabetic patients [41]. As GPIIbIIIa are common cellular adhesion receptors, it is very important to study their role in the specific mechanisms of fibrinogen-induced RBC aggregation.

The aim of the present work was to implement a combination of various optical techniques for a comprehensive study of the specific mechanisms of fibrinogen macromolecules interaction with the RBC membrane. Laser optical approaches have a great potential for a deep, thorough study of the biochemical and biophysical properties of blood [42]. In particular, laser aggregometry was successfully used to study microrheologic alterations of blood at various conditions accompanied with disturbances in protein content in plasma [22,43]. Flow cytometry is a well-known method for studying parameters of the interactions between different components of blood [44]. Optical trapping proved to be a very useful approach for studying peculiarities of RBC interaction, microrheology, and biomechanics [18,45,46,47,48] as well as for quantifications of a number of proaggregant macromolecules adsorbed on the surface of RBC [49]. In the present work, we implemented advanced optical techniques including the diffuse light scattering technique (laser aggregometry), flow cytometry, and optical (laser) tweezers combined with microfluidics for the assessment of novel features describing the interactions between blood plasma protein fibrinogen and the RBC membrane. We focused on the complex assessment of the characteristics of fibrinogen macromolecular interaction with the RBC membrane and verified its contribution to the mechanisms of RBC reversible aggregation. The obtained knowledge is of importance for understanding fundamental mechanisms of fibrinogen-induced RBC aggregation, which potentially discovers new opportunities for the correction of RBC hyper-aggregation conditions at various diseases.

## 2. Materials and Methods

### 2.1. Protocol of the Microscopic Studies of Alexa488-Labeled Fibrinogen Adsorption onto RBC Membrane

To study fibrinogen adsorption onto the membrane of a single RBC, the custom-built experimental setup, consisting of holographic optical tweezers (OT) combined with a fluorescence microscope and microfluidic system, was used. The detailed description of this facility is available in our previous works [49,50].

The schematic layout of the experimental setup is demonstrated in Figure S1. The microfluidic chip contains the incubation chamber, which is filled with the stock solution of fluorescent-labeled fibrinogen and RBC. This chamber is directly connected to a microchannel, which is constantly flushed with phosphate buffered saline (PBS). Flushing allows preventing the penetration of the fluorescent dye into the channel. During the measurements, the RBC are trapped by OT in the incubation chamber and moved deeply into the microchannel as far as possible (the final positioning is described in Figure S1 with a black dashed line circle; the whole positioning process is demonstrated in Supplementary Video S1). As the channel is constantly flushed with PBS (media without fibrinogen), the chemical equilibrium in this area is shifted to the processes of fibrinogen desorption from the RBC membrane. Immediately after the correct positioning of the RBC, the fluorescent microscopy regime is turned on, and the fluorescent signal measurements are performed. In such implementation, we are able to observe simultaneously the adsorbed by RBC membrane fluorescent labelled fibrinogen and the decay of the fluorescent signal from the cell caused by fibrinogen desorption.

Trapping the doublet of RBC using OT and moving it into the microfluidic channel allows for observations of the fluorescence of the labeled molecules adsorbed on the surface of the cell membranes. Optical traps were formed using a laser beam from the single-mode Nd:YAG laser (1064 nm, 1 W, Ventus, Laser Quantum, Stockport, United Kingdom) reflected by the parallel-aligned liquid crystal spatial light modulator (PAL-SLM, PPM X8267-15, Hamamatsu Photonics, Hamamatsu, Japan) and focused with a large numerical aperture oil immersion objective (NA 1.25, 60×, Nikon, Tokyo, Japan). The location of traps within the focal plane of the objective was controlled by PAL-SLM using software in the MatLab environment. Visual control of the trapped cells was done in the transmission configuration using the digital CMOS camera (Orca-Flash 4.0 V3, Hamamatsu Photonics, Hamamatsu, Japan). To be able to hold the RBC in the OT under the flow, the trapping laser beam power was set to 38 mW. At this magnitude, we did not observe significant alterations of the cell morphology under the laser trapping influence. The whole setup was based on the inverted microscope (Eclipse TE 2000, Nikon, Tokyo, Japan).

In this study, the Alexa-488 conjugated fibrinogen from human plasma (Thermo Fisher, F13191, Invitrogen, Carlsbad, CA, USA) was used. Alexa488–fibrinogen conjugates are widely used nowadays to study the interactions between blood plasma components and the blood cells membrane [51,52,53]. Fibrinogen labeled with Alexa488 fluorophore is described in [54] and is considered to be a functional analogue of native fibrinogen. In [32], such conjugates were directly used to study the interaction between fibrinogen and the CD47 receptor complex on the RBC membrane. The stock solution was prepared by dilution of the conjugate in PBS so that the final concentration of fibrinogen was 3 mg/mL. During this experiment, the RBC of a healthy male donor were obtained by the finger-pricking method using a sterile lancet. Right after the extraction, RBC were washed in PBS and incubated in fibrinogen–Alexa488 solution for 2 h at 37°C at 1% hematocrit (HCT). After the incubation, this solution with RBC was drawn into the microfluidic setup, and the measurements were performed.

### 2.2. The Protocol of Cells Preparation for the Measurements of Hydrodynamic Strength of RBC Aggregates Using Laser Aggregometry

Laser aggregometry experiments involved the blood drawn from the cubital veins of 5 healthy male donors. The blood was sampled into containers with ethylenediaminetetraacetic acid (Vacuette EDTA K3E tube, 1.8 mg/ 1 mL of blood, 4.5 mL). RBC were extracted by centrifugation of the whole blood during 10 min at 180 g (Eppendorf MiniSpin, Germany). Right after that, RBC were washed in PBS (Gibco, pH 7.4, Thermo Fischer Scientific, New York, NY, USA) 3 times (3000 g, 3 min). The autologous serum was prepared according to the standard protocol using a Vacuette 454071 tube with the clot activator.

To study fibrinogen-specific adsorption on the RBC membrane and the mechanisms of RBC aggregation, we investigated the influence of disintegrin eptifibatide (eptifibatide acetate, Sigma Aldrich, SML 1042, Merck, Darmstadt, Germany) on the hydrodynamic strength of RBC aggregates. Eptifibatide is known as a common GPIIbIIIa inhibitor. The stock solution of eptifibatide was prepared using PBS and subsequently diluted with PBS to achieve the desired concentrations. The choice of concentrations was made according to the previous studies corresponding to the typical values used in clinical practice during the intravenous delivery of the eptifibatide into the blood of the patient [31,33].

The protocol of studying the effects of eptifibatide on fibrinogen adsorption on the RBC membrane included the following procedure: washed RBC were incubated in PBS solution of human plasma fibrinogen (Sigma Aldrich, F3879, Merck, Darmstadt, Germany) at 3 mg/mL for 30 min at 37 °C at 50% hematocrit (HCT) in the presence of eptifibatide at various concentrations and without eptifibatide in the control sample. As the binding of fibrinogen to GPIIbIIIa requires the presence of millimolar concentrations of divalent cations [55], calcium chloride (CaCl2, Sigma Aldrich, C1016, Merck, Darmstadt, Germany) was added so that the final concentration of calcium was 1 mM. After the incubation, RBC were extracted and added into the autologous serum at 40% HCT for further aggregometry measurements. Additionally, to support our findings on the single cell level, the RBC sample was resuspended in autologous serum at 0.1% HCT to measure RBC aggregation forces using laser tweezers.

Recommendations for hemorheological laboratories developed by the international expert group created for hemorheological research standardization [56] were considered. The study was approved by the ethics committees of M.V. Lomonosov Moscow State University. All volunteers were informed on the purpose of the study and gave written informed consent in accordance with the Declaration of Helsinki.

### 2.3. Laser Aggregometry Measurements. Critical Shear Stress

In the present work, to estimate the changes of hydrodynamic strength of RBC aggregates, we used the critical shear stress (CSS) parameter—the minimum shear stress required to initiate the process of the forced disaggregation of RBC aggregates under the shear flow. Measurements of CSS were performed in the channel of the microchip utilizing laser aggregometer RheoScan AnD-300 (Rheomeditech, Seoul, Republic of Korea). The whole procedure of CSS measurements and the photograph of the microchip are provided in Figure S2. The detailed description of RheoScan AnD-300 functioning is available in the works of the developers group [57,58].

### 2.4. Optical Tweezers Measurements of Interaction Forces Between Individual RBC

**Figure 1:**
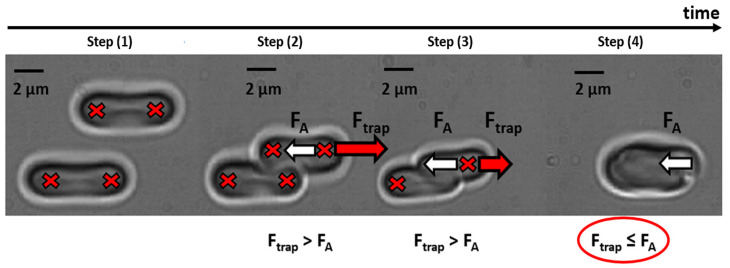
The step-wise protocol of the measurement of the aggregation force (F_A_) utilizing optical tweezers (OT). Step (1): Two different red blood cells (RBC) are trapped with laser tweezers (red crosses) with equal trapping force F_trap_ (red arrow points the direction of F_trap_); step (2): RBC are brought to a contact; step (3): the middle traps are switched off, and cells begin to interact with each other, tending to form an aggregate because of the existence of the force that drives the aggregation process (aggregation force F_A_, white arrow points in the direction of F_A_). However, they do not overlap, because the optical trapping prevents the aggregation (the condition F_trap_ >F_A_ is fulfilled). Starting from this moment, we begin to decrease the laser beam power (and, correspondingly, F_trap_). At some point, cells overlap (step (4)), because the trapping force is not sufficient to prevent the RBC aggregation (F_trap_ ≤ F_A_). As we know the laser beam power at this moment, we can measure F_A_.

Optical tweezers (OT) were used for measuring the changes of RBC interaction forces at cells spontaneous pair aggregation under the effects of GPIIbIIIa inhibition with eptifibatide. RBC interaction forces at spontaneous aggregation can be characterized with the aggregating force (F_A_) defined as a minimum force needed to prevent the cells from spontaneous aggregation and thus can be related to the hydrodynamic strength of aggregates. The method of F_A_ measurements using OT was introduced in our previous papers [50,59,60]. In the present work, we provide a brief step-wise protocol of the measurement of F_A_ using OT (Figure 1). An example of the single measurement is demonstrated in Supplementary Video S2.

At the initial time point (step 1 in Figure 1), two different RBC are trapped with four laser traps (red crosses in Figure 1) with equal trapping force F_trap_. After that, these two cells are brought to a contact (step 2 in Figure 1). In every measurement, the cross-section of their interfaces remained unchanged, which was controlled by two middle traps. Then, middle traps were simultaneously switched off (step 3 in Figure 1). Two cells intensively interact with each other (see Supplementary Video S2), but the remaining optical traps prevent them from complete overlapping, because the trapping force F_trap_ (red arrow in Figure 1) exceeds F_A_ (white arrow in Figure 1). Starting from this moment, we step-wisely decrease the laser power with a corresponding decrease in the trapping force. At some point (step 4 in Figure 1), the trapping force is not sufficient to prevent the cells from overlapping (F_trap_ ≤ F_A_). We measure the laser beam power and corresponding F_trap_ in this particular moment, assuming it equals the minimum force that is required to prevent the aggregation. The experimental chamber was a 500 µL reservoir made in an aluminum frame closed with glass covers from both sides to prevent the evaporation of the sample.

Prior to the measurements, the OT were calibrated to estimate the correlation between the F_trap_ and the beam power. To do that, we used the calibration procedure based on comparison of the F_trap_ with the viscous Stocks force. The whole algorithm is described in our previous work [61].

### 2.5. The Protocol of the Flow Cytometry Assay of the Specific Mechanisms of Alexa488-Labeled Fibrinogen Adsorption onto RBC Membrane

To perform the flow cytometry experiments, healthy male donor RBC were extracted by the finger-pricking method. Right after the extraction, RBC were washed in the buffer solution (140 mM NaCl, 2 mM CaCl2, 2.8 mM KCl, 4 mM MgCl2, 20 mM 4-(2-hydroxyethyl)-1-piperazineethanesulfonic acid (HEPES), 10 mM glucose; pH 7.4).

The staining of the RBC sample was performed as the following: first, 2 µL of washed RBC were resuspended in 10 µL of Alexa488-labeled fibrinogen solution (3 mg/mL) and incubated during 30 min at 37°C. After the incubation, 1 µL of this suspension was added to 500 µL of the buffer and measured on the cytometer. The control (not stained) RBC sample was prepared according to the same protocol, except the dilution of washed RBC was performed in 10 µL of non-labeled fibrinogen at 3 mg/mL.

The flow cytometry study of the effects of fibrinogen-specific binding inhibition involved the usage of four substances: eptifibatide; tirofiban; RGDS; fibrinogen binding inhibitor peptide. The stock solutions of tirofiban (tirofiban hydrochloride monohydrate, Sigma Aldrich, SML 0246, Merck, Darmstadt, Germany) and fibrinogen binding inhibitor peptide (Sigma Aldrich, Merck, Darmstadt, Germany) were prepared by the dilution of dry powders in dimethyl sulfoxide (DMSO) to achieve 25 and 1 mM concentrations, correspondingly. The stock solutions of eptifibatide (eptifibatide acetate, Sigma Aldrich, SML 1042, Merck, Darmstadt, Germany) and RGDS (Arg-Gly-Asp-Ser, Sigma Aldrich, A9041, Merck, Darmstadt, Germany) were prepared by dilution in PBS. The aliquots of the stock solutions of the substances were subsequentially diluted with the buffer solution to achieve desired concentrations involved in the experiment.

To evaluate the effects of the GPIIbIIIa inhibition on the adsorption of fibrinogen on an RBC membrane, prior to the staining, 2 µL of washed RBC were added into 100 µL of the solution of the inhibitor and incubated for 20 min at 37 °C. The concentrations of the inhibitors were as follows: [eptifibatide] 0.25 mM; [tirofiban] 2.5 mM; [RGDS] 5.0 mM; [fibrinogen binding inhibitor peptide] 0.125 mM. After the incubation, the RBC were sedimented by centrifuging (1 min at 180 g), the supernatant was changed with the 10 µL of fibrinogen–Alexa488 conjugate, and the staining was performed according to the same protocol described above.

To provide the non-specific binding control test, we studied the effects of GPIIbIIIa inhibitors on the sorption of fluorescein isothiocyanate (FITC)-labeled human serum albumin (HSA). The staining of washed RBC was performed by the dilution of 2 µL of cells sample in 10 µL of FITC-labeled HSA conjugate (at a concentration of 10 mg/mL) for 20 min at 37°C. The experimental conditions of the inhibition tests were the same.

Flow cytometry experiments were performed with the SH800S Cell Sorter system (Sony Biotechnologies, Tokyo, Japan) using an excitation source at 488 nm for rapid measurements of the fluorescent-labeled proteins, which were adsorbed on the surface of the RBC. That allowed us to estimate the adsorption of the fibrinogen and, moreover, to verify the effects of glycoproteins inhibition on Alexa488–fibrinogen adsorption. The fluorescent signal was evaluated in the FITC spectral channel (ex = 488 nm/ em = 525 (40) nm). FSC channel corresponds to forward light scattering. No less than 10^5^ events were detected in each experiment. The gating strategy based on the analysis of FSC diagrams was performed to distinguish the cluster of single RBC to exclude the doublets from the dataset. The example of the gating is provided in Figure S3.

### 2.6. Statistics and Data Presentation

To analyze the differences between the measured set of values in the experiments involving laser aggregometry and optical trapping, a standard T-test was implemented in the software package Statistica 12.6 (StatSoft, USA). To verify the statistical significance of the observed differences in flow cytometry data, the comparison between the data on the control subsample of cells and the subsample of RBC exposed to the inhibitors was carried out with the Kruskal–Wallis H-test using the Python programming language and NumPy and SciPy libraries. Time courses of fluorescent signal were obtained analyzing the initial images in the software package ImageJ. The mathematical analysis of the fluorescent experimental data was performed in the software package Origin Pro 2018 (OriginLab, USA).

## 3. Results

### 3.1. Microscopy Visualization of Fibrinogen Macromolecules Adsorption onto RBC Membrane: Role of Fibrinogen Adsorption and Desorption in the Process of RBC Aggregation

**Figure 2:**
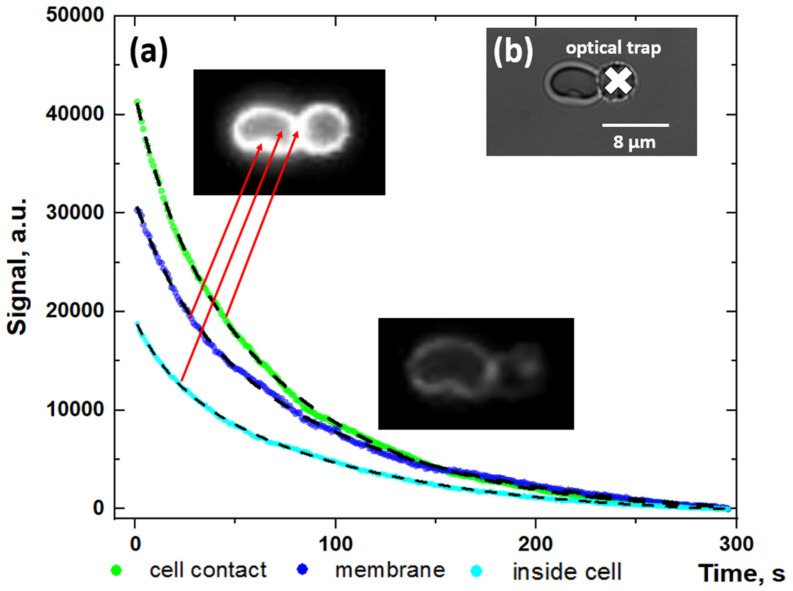
Fluorescent measurements results: (**A**) Time dependencies of the fluorescent signal of Alexa488-labeled fibrinogen adsorbed on the membrane of the doublet of RBC in different regions of the cell (linear scale); (**B**) Image of the doublet of RBC, trapped using optical tweezers in the microchannel. The white cross points to the optical trap position. Red arrows point to the regions of fluorescent signal measurements. Dashed curves present the results of fitting of the data using the exponential decay function with mean decay time τ ≈ 70.2 ± 5.5 s. Fits with the mono-exponential decay function in the semi-log scale are available in the Supplementary Materials (Figure S4). Parameters of the fitting are presented in the Supplementary Table S1. The whole process is demonstrated in Supplementary Video S3 (video accel. x20 times).

We visualized the adsorption of fibrinogen macromolecules onto the RBC membrane with the usage of optical tweezers combined with a microfluidics setup. The observed signal distribution demonstrates the adsorption of Alexa488-conjugated fibrinogen on the cellular membrane (please see Supplementary Video S3). Figure 2A demonstrates the time courses of the fluorescent signal from different regions in the RBC doublet, which was incubated with fluorescent-labeled fibrinogen. Figure 2B shows the doublet of RBC in which one cell is trapped by the laser tweezer during the measurements. Analysis of the time dependencies of a fluorescence signal in linear (Figure 2A) and semi-log (Figure S4B) scales demonstrates that fitting with the mono exponential decay function can be performed, revealing the process with the relaxation time (τ) of the fluorescent signal from 64.0 ± 0.4 s to 74.3 ± 0.7 s, depending on the region of the fluorescence measurements. The different signal values in different areas of the RBC doublet can be explained by the signal defocusing during the measurements. Parameters of the fitting are presented in the Supplementary Table S1.

**Figure 3:**
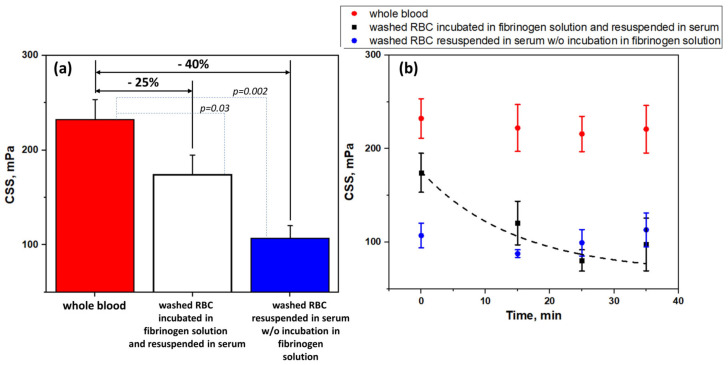
Hydrodynamic strength of RBC aggregates in terms of critical shear stress (CSS): (**A**) In different samples: whole blood; washed RBC, pre-incubated in fibrinogen solution (3 mg/mL) and resuspended in autologous serum; washed RBC, resuspended in autologous serum without preliminary incubation in fibrinogen solution; (**B**) The time dependence kinetics of CSS of RBC aggregates in different samples: whole blood; washed RBC, pre-incubated in fibrinogen solution (3 mg/mL) and resuspended in autologous serum; washed RBC, resuspended in serum without preliminary incubation in fibrinogen solution. Black dashed line represents the fitting with mono-exponential decay function. N = 10, mean ± SD. *p* was calculated using standard T-test.

Figure 3 presents the results of a study of the role of fibrinogen adsorption and desorption in RBC aggregation. Figure 3A shows the role of fibrinogen adsorption. CSS as a characteristic of the hydrodynamic strength of RBC aggregates was measured in different samples: (1) whole blood; (2) washed RBC pre-incubated in human plasma fibrinogen solution (3 mg/mL) and resuspended in autologous serum; (3) washed RBC resuspended in autologous serum without pre-incubation in fibrinogen solution. As it is seen in Figure 3A, washing RBC and further resuspension in autologous serum (CSS = 142 ± 25 mPa) led to the 40% decrease of CSS in comparison with the whole blood (CSS = 232 ± 30 mPa), while preliminary incubation in the fibrinogen solution at physiological fibrinogen concentration (3 mg/mL) led to a 25% decrease (CSS = 174 ± 21 mPa). It means that the adsorption of fibrinogen, which took place during the preliminary incubation in fibrinogen solution after the washing and before inserting cells into the serum, supplied a 15% increase in the hydrodynamic strength of RBC aggregates.

We assume that the hydrodynamic strength of fibrinogen-induced aggregation of RBC should decrease due to the desorption of fibrinogen macromolecules from cells membrane when cells are put into the media without fibrinogen. To estimate this decrease, we performed several time tests (the results are demonstrated in Figure 3B). We measured the time dependence of CSS of the washed RBC, which were resuspended in autologous serum after pre-incubation in the fibrinogen (3 mg/mL) solution (Figure 3B, black dots). It was found that the CSS decreased exponentially to some minimum level, while it was not abolished completely. Meanwhile, the CSS of RBC in the whole blood (Figure 3B, red dots) and autologous serum (Figure 3B, blue dots) did not significantly change in the same time period. The time kinetics of CSS of washed RBC pre-incubated in fibrinogen and resuspended in serum can be fitted with the mono-exponential decay function with the relaxation time τ ≈ 480 s (blacked dashed line in Figure 3B).

### 3.2. Mechanisms of Fibrinogen Macromolecules Adsorption onto RBC Membrane Revealed by Flow Cytometry: Effects of Glycoproteins Inhibition and RBC Ageing

**Figure 4:**
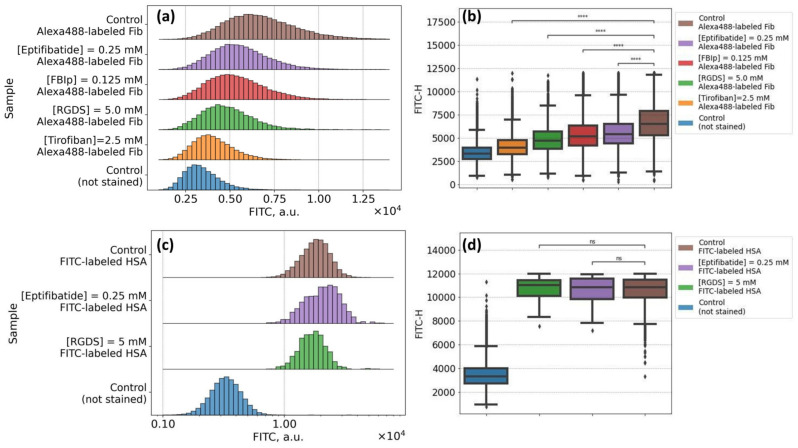
Flow cytometry results of the studying of interaction between fibrinogen macromolecules and the RBC membrane: (**A**,**B**) Specific binding test: changes observed in the fluorescein isothiocyanate (FITC-H) spectral channel for RBC samples, stained with Alexa488-labeled fibrinogen (3 mg/mL), preliminarily incubated in the solution of glycoproteins IIbIIIa inhibitors [eptifibatide] 0.25 mM; [fibrinogen binding inhibitor peptide, FBIp] 0.125 mM; [RGDS] 5 mM; [tirofiban] 2.5 mM; (**C**,**D**) Non-specific binding test: changes in FITC-H channel for RBC, preliminary incubated in glycoproteins IIbIIIa inhibitors solution and stained with FITC-labeled human serum albumin (10 mg/mL). **** *p* < 10^−5^ was calculated using the Kruskal–Wallis H-test; ns: non-significant.

Figure 4A,B demonstrate the results of the flow cytometry assay of the sorption of Alexa488-labeled fibrinogen onto the RBC membrane. We were able to observe the significant increase in the signal values in the FITC-H spectral channel for the stained RBC (brown-colored diagrams) in comparison with the autofluorescent signal of intact RBC (blue-colored diagrams). Such an increase is governed by the binding of Alexa488-labeled fibrinogen by washed RBC. The preliminary exposition of RBC to the glycoproteins inhibition led to the clearly observable decrease (in comparison with stained control RBC sample) in signal in the FITC-H channel for all inhibitors involved. The most dramatic decrease was observed for tirofiban (Figure 4A,B, orange diagrams), while the effects of other inhibitors (eptifibatide, RGDS, and fibrinogen binding inhibitor peptide) were less yet significant. Based on this result, we may assume that an interaction between fibrinogen and RBC membrane is at least partially specific, and glycoproteins IIbIIIa can serve as potential specific binding sites, which corresponds to the data published by other groups.

In support of our findings, we performed a non-specific binding control test by evaluation of the effects of glycoproteins IIbIIIa inhibition on the sorption of FITC-labeled human serum albumin (HSA). The results are presented in Figure 4C,D. We can see the increase in the signal in the FITC-H channel for washed RBC stained with FITC-HSA conjugate in comparison with intact non-stained RBC samples demonstrating the adsorption of albumin onto the cellular membrane due to the albumin capacity of binding to lipids within the RBC membrane [62]. That also corresponds to the data in the literature on the interactions between the RBC membrane and serum albumin [63,64]. The preliminary incubation of RBC in the presence of glycoproteins IIbIIIa inhibitors (eptifibatide, violet diagrams in Figure 4C,D; or RGDS, green diagrams in Figure 4C,D) did not show any influence on the staining. That suggests that the decrease in fluorescent channel, which was observed for fibrinogen–Alexa488 staining, is governed by the inhibition of the specific binding via motifs in glycoproteins IIbIIIa on the surface of RBC.

**Figure 5:**
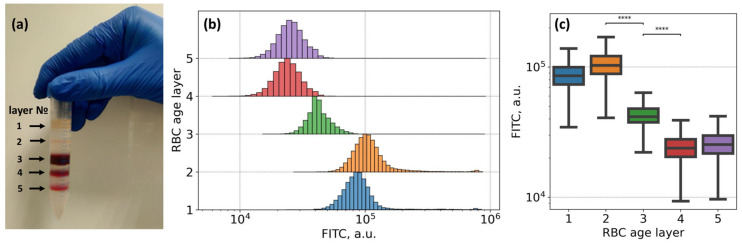
Flow cytometry results demonstrating the dependence of membrane adsorption of Alexa488-conjugated fibrinogen macromolecules on the age of RBC: (**A**) RBC age layers distribution using density separation into five fractions: layers from 1 to 5, youngest cells are on top (layer 1) and oldest are on the bottom (layer 5); (**B**,**C**) Changes observed in the fluorescein isothiocyanate (FITC-H) spectral channel for RBC, sampled from different layers (i.e., RBC at different age), stained with Alexa488-labeled fibrinogen (3 mg/mL). **** *p* < 10^−6^ was calculated using Kruskal–Wallis H-test with Bonferroni correction. Age-layering of the fresh RBC was obtained upon Percoll gradient separation.

As an additional control for our flow cytometry study of fibrinogen interaction with the RBC membrane, we performed a set of cytometric measurements of Alexa488-labeled fibrinogen staining of RBC of different ages. It is known [65] that during aging, the number of fibrinogen molecules bound to the RBC membrane significantly decreases. According to this, we assessed the changes in the fluorescence signal from Alexa488-labeled fibrinogen bound by RBC of different ages. As far as the progressive loss of cell area and cellular dehydration are both characteristic features of RBC senescence [66], it is possible to segregate RBC by age using density separation [67,68]. RBC age separation was carried out according to the following protocol: 3 times washed RBC diluted with PBS in a 1:1 ratio was centrifuged (4000 g, 30 min, 4°C) over Percoll gradients (densities varying from 1.085 to 1.122 g/mL). This provided five fractions (layers): the youngest to the top and the oldest to the bottom (Figure 5A). RBC of different fractions were washed in PBS 3 times and then resuspended in PBS for the following staining with Alexa488–fibrinogen conjugate.

The results of the flow cytometry assay of the RBC of different ages, stained with Alexa488–fibrinogen conjugate, are presented in Figure 5B,C. We observed a statistically significant (Kruskal–Wallis test with Bonferroni correction, *p*-value < 10^−6^) difference between all fluorescence intensities of all subsamples of RBC of different age layers, yet the most prominent and practically significant shift was observed between the fluorescence intensity of 2nd and 3rd, and 3rd and 4th cell layers (marked as **** in Figure 5C). It means that younger fractions of erythrocytes (layers 1 and 2) are able to bind significantly more fibrinogen than cells of 3, 4, or 5 layers, which predominately consist of older erythrocytes.

### 3.3. Effects of Glycoproteins IIbIIIa Inhibition on RBC Aggregation

**Figure 6:**
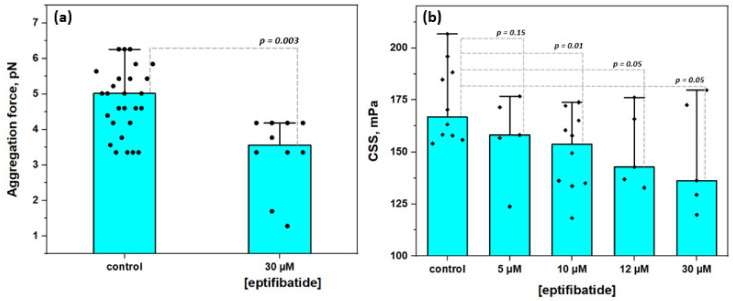
The effects of glycoproteins IIbIIIa inhibition with eptifibatide on the aggregation of RBC: (**A**) Changes in RBC aggregation force; (**B**) The dose-dependent decrease in hydrodynamic strength of RBC aggregates (in terms of critical shear stress, CSS). Mean values ± SD are demonstrated. *p* was calculated using standard T-test.

Figure 6 demonstrates the results of the microrheologic study of the effects of glycoproteins IIbIIIa inhibition with eptifibatide on RBC aggregation induced by the pre-incubation of cells in fibrinogen (3 mg/mL) solution. Control samples represent RBC resuspended in autologous serum after pre-incubation in fibrinogen (3 mg/mL) solution without the addition of eptifibatide. Figure 6A shows the decrease in RBC aggregating force F_A_ from 4.8 ± 0.9 pN in the control sample to 3.1 ± 0.2 pN at [eptifibatide] = 30 µM. It means that the presence of eptifibatide in fibrinogen solution during the preliminary incubation stage led to the decrease in the amount of fibrinogen absorbed on the membrane, therefore reducing the bridging-related aggregation forces. Figure 6B demonstrates the similar trend in hydrodynamic strength of RBC in terms of CSS values: we observed a clear concentration-dependent decrease in CSS from 174.2 ± 18.2 mPa in the control sample up to 128.3 ± 20.4 mPa when 30 µM of eptifibatide was added to the preliminary fibrinogen incubation solution. Thus, the overall maximum inhibition effect was a 15% decrease in the RBC aggregates hydrodynamic strength in comparison with the control values, which is in agreement with the previous results obtained by other methods [34].

## 4. Discussion

A combination of optical trapping with the microfluidics technique allowed clearly demonstrating the fibrinogen macromolecular adsorption onto the membrane of RBC. We examined the distribution of Alexa488-labeled fibrinogen macromolecules on the membrane of RBC at different concentrations of fibrinogen in the incubation solution (Figure S5). We can see that the time course of the fluorescent signal does not depend on the concentration of fibrinogen in the physiological range of concentrations (2–3 mg/mL).

We were able to demonstrate the role of fibrinogen adsorption in the process of RBC reversible aggregation. The decrease in the hydrodynamic strength of RBC aggregates of washed RBC resuspended in autologous serum in comparison with that for RBC pre-incubated in fibrinogen solution strongly points to the contribution of fibrinogen adsorption onto RBC membrane in the strengthening of RBC aggregates. The preliminary incubation of washed RBC in fibrinogen solution provides a 15% increase in RBC aggregates strength. Afterwards, this difference disappears due to the fibrinogen desorption process. Being inserted into the media completely deprived of fibrinogen, after some period of time, RBC aggregates become less strong, reaching level when the CSS is at its minimum. We assume this state corresponds to the equilibrium when all fibrinogen is desorbed from the membranes of all RBC in the sample and yielded into the media.

Our results also indicate the presence of fibrinogen-specific binding sites on the RBC membrane. The inhibition of the fibrinogen-specific binding resulted in the significant weakening of the interaction between fibrinogen macromolecules and the erythrocytes membrane. First of all, based on the results of flow cytometry that provide the data on the ensemble of a huge number of cells, we can conclude that the preliminary exposition of RBC to fibrinogen-specific binding inhibitors leads to a significant decrease in the amount of fibrinogen adsorbed on the RBC membrane (Figure 4A,B). In particular, the inhibition of glycoproteins IIbIIIa (GPIIbIIIa) with tirofiban, eptifibatide, and RGDS led to a reliable decrease in the adsorption of the fluorescent-labeled fibrinogen. Secondly, these results support the obtained data on the microrheological effects of GPIIbIIIa inhibition: we observed a clear decrease in RBC aggregation on the single cell level (aggregating forces, Figure 6A) or on the level of RBC suspension at a high hematocrit level (CSS values, Figure 6B) when the GPIIbIIIa inhibitor (eptifibatide) was presented in the experimental solution. Our preliminary results show that the in vitro administration of eptifibatide into the blood samples of patients with arterial hypertension leads to a decrease in the parameters of RBC aggregation (the data on the changes in RBC aggregation index and CSS of arterial hypertension patients under effects of eptifibatide are shown in Figure S6). Glycoproteins activation remains one of the major problems for clinical blood-contacting devices, materials, and interfaces [69,70]. Platelets and RBC intensively interact with any artificial material surface, which creates critical risks of hyper-coagulation. As GPIIbIIIa are common cellular adhesion proteins, it is very important to be able to control their functioning to prevent RBC hyper-aggregation.

We also observed that the complete abolishment of RBC aggregation was not obtained even at the full inhibition of GPIIbIIIa. That can be explained by several reasons. The first one is that the fibrinogen molecular bridging is not the only contributor to the RBC aggregation [8,13,71]. The reversible character of the inhibition effect [31,37] or a simultaneous existence of inactive and active conformations of glycoproteins receptors in the membrane [35] can also be the point. Another possible explanation is that fibrinogen-specific binding sites on the RBC membrane are not completely glycoproteins but related structures, which possess several recognition sequences complementary to the ones in fibrinogen. This hypothesis is supported by the results presented in [31], demonstrating that the fibrinogen receptor on the RBC membrane is not as strongly influenced by calcium as in case of platelets. Studies of the mechanisms of fibrinogen-induced RBC aggregation are also complicated by the fact that the plasma fibrinogen represents a mixture of different isoforms of γ chains, resulting in the heterogeneity of fibrinogen fraction in charge and sizes [72]. In the work [73], it was demonstrated that the γ’/γ’-dimeric fibrinogen has an increased binding force to RBC in comparison with γAγA fibrinogen, which is composed of 85–90% total plasma fibrinogen.

Additionally, besides glycoproteins IIbIIIa, mature RBC express a number of adhesion molecules (CD36, CD44, CD47, CD58) and others that have potential adhesion properties (intercellular adhesion molecules (ICAM-1 (CD54) and LW/ICAM-4 (CD242)), basal cell adhesion molecules (B-CAM/Lu(CD239)), VLA-4 (integrin complex α4β1 (CD49d/CD29), CD99, CD108 and CD147) [27,74,75,76]. However, only some of them can be related to the specific mechanisms of fibrinogen-induced RBC aggregation. CD47 is an integrin-associated transmembrane protein expressed on the membrane of erythrocytes as a mechanism of phagocytosis prevention [77]. Erythrocytes with low CD47 expression, or the expression of CD47 with structural abnormalities caused by genetic alterations or oxidative stress, are taken from the circulatory system by the spleen macrophages. CD47 is also known as a molecular target for several proteins such as thrombospondin, laminin, and fibronectin [78]. Taking the properties of the CD47 into account, De Oliveira et al. [32] demonstrated that anti-CD47 monoclonal antibodies significantly decreased the fibrinogen binding by RBC. In addition, in their work, authors showed that younger RBC are able to bind fibrinogen more effectively, which circumstantially points to the involvement of CD47 in the specific binding of fibrinogen, as CD47 expression is strongly associated with RBC aging. The authors stated the necessity of further experiments on the study of fibrinogen interactions with RBC to determine the role of integrin-associated protein complexes in more detail. CD147 (basigin; extracellular matrix metalloproteinase inducer (EMMPRIN)) is a main determinant of the blood group antigen Ok system. It is considered as an adhesion molecule in several pathological conditions, such as malaria during *P. falciparum* invasion [79] and during the development of SARS-CoV-2, which is accompanied with acute inflammation and corresponding alterations in RBC aggregation and microrheology [80].

Intercellular adhesion molecules (ICAM) represent a family of integrin molecular complexes of different types, which play a significant role in the mechanisms of the interactions between different types of blood cells. In [81], the authors indicate that the sickle-cell RBC of patients after shunting do not directly interact with ICAM-1 on endothelium cells; rather, fibrinogen, bound by the RBC membrane, triggers the adhesive connection by forming an intercellular bridge without any additional molecular intermediate. ICAM-4 is expressed on the membrane of erythroid cells, giving them an ability to interact with several types of integrins expressed on other types of blood cells, i.e., white blood cells [82]. In [75], it was demonstrated that GPIIbIIIa can serve as a receptor for ICAM-4, and their binding can be the mechanism driving RBC interactions with platelets during the stabilization of the clot formation. As it can be seen, various adhesion molecules, including CD47, CD147, and ICAM, can implement the bridging constituent in the mechanisms of fibrinogen-induced aggregation of RBC. Therefore, this can explain why the RBC aggregation cannot be abolished completely, even at high concentration of GPIIbIIIa inhibitor.

The strong dependence of fibrinogen interaction with the RBC membrane on the age of erythrocytes was clearly demonstrated in our flow cytometry experiments (Figure 5). These findings are supported by works of other groups who evaluated the age-related alterations of the RBC membrane structural compound. Namely, progressive loss of the cell area and cell dehydration as characteristic features of RBC aging [66] may be responsible for the impairments of fibrinogen binding. Age-related biochemical changes in RBC membrane components and organization, such as alterations in the phospholipids composition [83], receptors conversion into non-active form [84], structural changes, and modifications of membrane protein complexes (e.g., phosphorylation of band 3 complexes [85]), are mostly caused by oxidative stress and thus play a role in the regulation of the interaction between cells and blood plasma proteins, including fibrinogen. Therefore, the variations of fibrinogen–erythrocytes-specific interactions in relevance to the age of erythrocytes should be considered in medical practice in the protocols of the treatment of hemorheological disorders, during blood storage and transfusion procedures.

RBC aggregation was also not abolished after a long period of incubation in fibrinogen-depleted media (i.e., serum). One of the possible explanations is that serum contains several proaggregant macromolecules. These macromolecules dominantly consist of globulins fractions namely immunoglobulins [71]. Several other proteins such as ceruloplasmin, haptoglobin, and α2-macroglobulin also demonstrate proaggregant effects [12,86]. The effect on RBC aggregation of other serum macromolecules such as albumin and C-reactive protein is controversial [87,88]. Another explanation is the duality of the RBC aggregation mechanism. Two possible mechanisms of RBC aggregation are proposed and widely investigated nowadays: molecular depletion-mediated interaction [10] and the bridging-induced interaction caused by the adsorption of macromolecules on the membranes of interacting interfaces [89]. According to the recent conceptions, both mechanisms are physiologically possible. Importantly, the kind of aggregation inducer is not limited to fibrinogen [87,90], and the type of particles to be aggregated is not limited to RBC [91]. The combination of synthetic macromolecules and liposomes can also induce aggregation without the involvement of the specific binding sites [91]. The hydrodynamic radius and the concentration of macromolecules decide the presence or the absence of particles in the aggregation process. Moreover, the complex synergetic effects of macromolecules of different weight and structure influence the aggregation of the particles in a very complicated way [71,92].

## 5. Conclusions

In the present work, we do not favor any aggregation mechanism, and our focus was the study of the features of the fibrinogen macromolecules adsorption on the cell membrane from the point of view of RBC as interacting bio-interfaces. Relying on the data on the existence of the mechanisms of fibrinogen-specific recognition by RBC [27,28,30,31,32,93] and on our own results [33,34,94], we assume that the interaction of fibrinogen with the RBC membrane has a specific character and GPIIbIIIa can serve as fibrinogen binding sites. According to this suggestion, the presence of the GPIIbIIIa inhibitors reduces the amount of adsorbed fibrinogen, leading to a decrease in the hydrodynamic stability of RBC aggregates (the thesis is illustrated in Figure S7). The role of macromolecules’ adsorption onto the RBC membrane is still not sufficiently well studied despite its significance in clinical hemorheology. Further studies of the functioning of GPIIbIIIa receptors on the RBC membrane in the mechanisms of disturbance in the RBC aggregation and its correction with the help of these receptor inhibitors are also required. For this purpose, non-invasive optical techniques, especially optical trapping, proved to be a very useful and innovative tool, which can be extremely helpful in the investigation of the fundamental properties of structural dynamic processes regulating the effectiveness of blood microcirculation.

## Supplementary Materials

The following are available online at https://www.mdpi.com/2218-273X/10/10/1448/s1, Figure S1: Schematic layout of the laser tweezers combined with microfluidic chip and fluorescence microscopy for measuring Alexa488-labeled fibrinogen macromolecules adsorption on the red blood cells membrane. Figure S2: Laser aggregometry measurements description: (a) typical time dependence of the intensity of laser light back scattered from the blood sample in the microchannel under the shear stress. The position of the maximum of light intensity curve characterizes critical shear stress (CSS); (b)–the image of microfluidic flow chamber with the microchannel for aggregometry measurements using RheoScan AnD-300; (c)–typical view of the result curve. Figure S3: Flow cytometry assay protocol: gating strategy for determination of the cluster of single RBC based on the values of forward light scattering (FSC). Figure S4: Fitting of the fluorescence signal time courses with mono-exponential decay functions in linear (a) and log scale (b). Figure S5: Time dependencies of the fluorescent signal of Alexa488-labeled fibrinogen adsorbed on the RBC membrane at different concentration of fibrinogen (linear scale). Fitting of the fluorescence signal time courses with mono-exponential decay functions. Images demonstrates the distribution of the signal at relaxation time τ. Figure S6: The effects of eptifibatide on the blood microrheological parameters of the patients with arterial hypertension measured in vitro using RheoScan AnD-300. Figure S7: The graphical illustration of the mechanisms of involvement of fibrinogen-specific binding sites in RBC reversible aggregation. Table S1: Parameters of the fitting of fluorescence signal curves in different regions of interest (ROI) on the surface of RBC doublet with mono-exponential function. Fitting performed in Origin 2018 software. Video S1: Positioning of the RBC in the channel of the microfluidic setup using optical tweezers for the measurements of the adsorption of the fibrinogen labeled with Alexa488. Video S2: Demonstration of the approach of RBC aggregation force (FA) measurement using optical (laser) tweezers. Video S3: Fluorescence signal distribution visualized on the doublet of RBC demonstrating the adsorption of Alexa488-conjugated fibrinogen on the cellular membrane.
